# Parkinson’s disease with anxiety: clinical characteristics and their correlation with oxidative stress, inflammation, and pathological proteins

**DOI:** 10.1186/s12877-024-04854-0

**Published:** 2024-05-16

**Authors:** Tenghong Lian, Weijiao Zhang, Danning Li, Peng Guo, Mingyue He, Yanan Zhang, Jinghui Li, Huiying Guan, Wenjing Zhang, Dongmei Luo, Weijia Zhang, Xiaomin Wang, Wei Zhang

**Affiliations:** 1 Center for Cognitive Neurology, Department of Neurology, Beijing Tiantan Hospital, Capital Medical University, Beijing, 100070 China; 2 Department of Neurology, Beijing Tiantan Hospital, Capital Medical University, Beijing, 100070 China; 3 Department of Blood Transfusion, Beijing Tiantan Hospital, Capital Medical University, Beijing, 100070 China; 4 Department of Physiology, Capital Medical University, Beijing, 100069 China; 5 Beijing Tiantan Hospital, China National Clinical Research Center for Neurological Diseases, Capital Medical University, Beijing, 100070 China; 6 Center of Parkinson’s Disease, Beijing Institute for Brain Disorders, Beijing, 100053 China; 7 Beijing Key Laboratory on Parkinson Disease, Beijing, 100053 China

**Keywords:** Parkinson’s disease_1_, Anxiety_2_, Clinical characteristics_3_, Oxidative stress_4_, Inflammation_5_, Pathological proteins_6_

## Abstract

**Objective:**

This study was performed to explore the differences in the clinical characteristics and oxidative stress indicators, inflammatory factors, and pathological proteins in serum between Parkinson’s disease (PD) with anxiety (PD-A) and with no anxiety (PD-NA) patients, and further correlations among clinical characteristics and above variables were analyzed in PD-A and PD-NA groups.

**Methods:**

A total of 121 patients with PD were enrolled in this study and assessed by the Hamilton Anxiety Scale (14 items) (HAMA-14). These patients were divided into PD-A and PD-NA groups according to a cut-off point of 7 of HAMA-14. Demographic variables were collected, and clinical symptoms were assessed by multiple rating scales. The levels of free radicals, inflammatory factors, and pathological proteins in serum were measured by chemical colorimetric method and enzyme-linked immunosorbent assay (ELISA). The differences of above variables were compared between PD-A and PD-NA groups, and the correlations of clinical symptoms with the abovevariables were analyzed in PD-A and PD-NA groups.

**Results:**

The frequency of PD-A was 62.81%. PD-A group exhibited significantly impaired motor dysfunction and multiple non-motor symptoms, including fatigue, sleep behavior disorder, restless leg syndrome and autonomic dysfunction, and dramatically compromised activities of daily living compard with PD-NA group. PD-A group displayed prominently increasedlevels of hydroxyl radical (·OH) and tumor necrosis factor (TNF)-α, and a decreased nitric oxide (NO) level in serum compared with PD-NA group (*P*<0.001, *P* = 0.001, *P*= 0.027, respectively). ·OH, NO, and TNF-α were identified as the risk factors of PD-A (OR = 1.005, *P* = 0.036; OR = 0.956, *P* = 0.017; OR = 1.039, *P* = 0.033, respectively). In PD patients, HAMA-14 score was significantly and positively correlated with the levels of ·OH and TNF-α in serum (*P*<0.001, *P* = 0.002, respectively). In PD-A group, ·OH level was significantly and negatively correlated with Aβ_1−42_ level, while TNF-α level was significantly and positively correlated with P-tau (S396) level in serum.

**Conclusions:**

The frequency of PD-A is high. PD-A patients present more severe motor dysfunction and multiple non-motor symptoms, and poorer activities of daily living. The increased levels of ·OH and TNF-α levels and the decreased NO level in serum are all associated with more severe anxiety in PD patients.Findings from this study may provide in-depth insights into the clinical characteristics, underlying mechanisms of PD-A, and potential correlations among anxiety, oxidative stress, inflammation, and cognitive decline in PD patients.

## Background

As a highly prevalent neurodegenerative disease after Alzheimer’s disease (AD), Parkinson’s disease (PD) mainly manifests as rest tremor, bradykinesia, rigidity, and posture and gait abnormalities. Importantly, PD patients have a variety of non-motor symptoms, such as olfactory dysfunction, anxiety, depression, sleep disorders, fatigue, autonomic dysfunction, and cognitive impairment. It has been demonstrated that non-motor symptoms in PD patients significantly reduce the activities of daily living (ADL) and robustly increase the burden of caregivers. Among various non-motor symptoms, depression, a very common mood disturbance of PD, has been profoundly investigated. However, anxiety, another frequently observed mood disturbance with a high incidence of closing to 60% [1], has not been sufficiently explored.

As reported in previous studies, α-synuclein is highly correlated with multiple non-motor symptoms of PD. For example, α-synuclein level in cerebrospinal fluid (CSF)/serum was significantly elevated in PD patients with apathy, fatigue or probable rapid eye movement sleep behavior disorder (PRBD) [2–4]. Contrary to α-synuclein, β amyloid (Aβ) _1−42_ level in CSF in PD patients with fatigue was evidently decreased. Aβ_1−42_ and phosphorylated tau (P-tau) [5] are the correspondent major components of neuroinflammatory plaques and neurofibrillary tangles in the brains of AD patients, and these two pathological proteins have also been observed in the brains of PD patients. According to previous studies, the levels of P-tau (S396) and T-tau in CSF were significantly elevated in PD patients with cognitive impairment [6, 7]. Interestingly, it has been proved that α-synuclein and pathological proteins of AD promote the deposition of each other. Although the “cross-seeding” hypothesis is widely recognized, there are few studies on the correlation between PD-A and the pathological proteins mentioned above.

Oxidative stress plays a crucial role on the development of PD. The involvement of oxidative stress in non-motor symptoms of PD has been observed in previous studies. In PD patients with apathy, the levels of ·OH and hydrogen peroxide (H_2_O_2_) in CSF were significantly elevated [2]. In PD patients with PRBD, NO level in CSF was also drastically elevated [4]. Moreover, increasing evidence suggests that inflammation in brain characterized by microglial over-activation promotes the development and progression of PD. It was found that microglia were over-activated in both PD patients and animal models, and hence inflammation is considered a pivotal mechanism of PD [8]. Activated microglia release numerous inflammatory factors, including tumor necrosis factor (TNF)-α, interleukin (IL)-1β, prostaglandin (PG) E_2_,and interferon (INF)-γ [9]. In previous investigations, inflammation has been linked to non-motor symptoms of PD [10]. In PD patients with PRBD, the levels of IL-1β and TNF-α in CSF were significantly elevated [4]. In PD patients with depression, TNF-α level in CSF was significantly higher than those without depression. In PD patients with cognitive impairment, the Montreal Cognitive Assessment (MoCA) score was significantly and negatively correlated with IL-6 level in CSF [6].

Both free radicals and inflammatory factors can further accelerate the formations and depositions of pathological proteins, such as α-synuclein, Aβ, and P-tau, leading to progressive degeneration and death of neurons. In PD patients with apathy, H_2_O_2_ level was positively correlated with α-synuclein level in CSF, suggesting that the excessively elevated α-synuclein in brain might be related to PD with apathy through oxidative stress [2]. However, there are few studies on the correlations of PD-A with free radicals and inflammatory factors in serum, as well as the correlations of free radicals and inflammatory factors in serum with pathological proteins

.

In this study, demographic variables of PD patients were collected, andanxiety, disease severity, and motor symptoms of PD patients were assessed by multiple professional rating scales. The levels of free radicals, including NO, H_2_O_2_, and ·OH, inflammatory factors, including IL-1β, IL-6, TNF-α, PGE_2_, and INF-γ, and pathological proteins, including α-synuclein, Aβ_1−42_, P-tau (T181), P-tau (S199), P-tau (T231), P-tau (S396), and total tau (T-tau),in the serum from PD patients were measured by using chemical colorimetric method and ELISA. The above variables were compared between PD-A and PD-NA groups, and the correlations among the above variables were analyzed in PD-A group.

## Methods

This cross-sectional study was designed to explore the clinical characteristics and their correlations with oxidative stress, inflammation, and pathological proteins in patients with PD-A.

### Ethics statement

This study was approved by the Review Board of Beijing Tiantan Hospital, Capital Medical University, and written informed consents were obtained from all participants.

### Participants

#### Inclusion criteria

The diagnosis of patients with PD was made according to the diagnostic criteria for PD of the 2015 International Parkinson and Movement Disorders Society (MDS) [11], with positive results from dopamine transporter-positron emission computed tomography.

PD-A was diagnosed based on the diagnostic criteria for PD patients with anxiety during the “off” period set by the Chinese Society of Neuropsychology and Behavioral Neurology [12]. As recommended by PD-A diagnostic criteria, HAMA-14 [13] was used to assess anxiety symptoms,covering both psychic anxiety (mental agitation and psychological distress) and somatic anxiety (physical complaints related to anxiety) for PD patients. Based on that, the clinical characteristics of patients with PD-A could be clearly and explicitly reflected. Patients with a total score of HAMA-14>7 or ≤ 7 were assigned to PD-A and PD-NA groups, respectively [14].

#### Exclusion criteria

Those who met the following criteria were excluded, including (1) patients with other central nervous systemic diseases; (2) patients with severe systemic diseases; (3) patients with secondary anxiety caused by hyperthyroidism, hypertension, coronary heart disease, and other physical diseases; (4) patients with psychopathological states, such as hallucination, delusion, obsessive-compulsive disorders, hypochondriac disorders, and phobia; (5) patients with infectious and immunological diseases; (6) patients with the administration of anti-anxiety drugs; (7) patients with withdrawal reactions resulted from stimulant overdose, hypnotic sedative, or anti-anxiety drugs.

According to the above criteria, a total of 121 PD patients were consecutively recruited from Beijing Tiantan Hospital, Capital Medical University.

### Clinical assessment

Anti-PD drugs were suspended for 12–14 h under proper conditions, and longer time was inappropriate after the assessment of our ethical committee. Since the medical washout period should be three times of the t1/2 of anti-PD drugs, subjects taking drugs with long t1/2, including levodopa controlled release, pramipexole, and piribedil controlled release, were excluded from this study.

#### Collections of demographic variables and medical information

Demographic variables, including gender, age, age of onset, and education level, and medical information, including disease duration, side of onset, and anti-PD therapy, including the types of drugs, levodopa equivalent daily dose (LEDD), and the duration of taking drugs, of PD patients were collected.

#### Assessments of motor functions

The severity of PD was assessed by using Hoehn-Yahr (H-Y) stage. Based on that, the severity of PD was classified into stages 0, 1, 2, 3, 4, and 5, respectively.

Motor symptoms, including rest tremor, bradykinesia, rigidity, and posture and gait abnormalities, were assessed by Unified Parkinson’s Disease Rating Scale (UPDRS) III during the “off” period to minimize the influence of medications on the assessments of motor symptoms. The higher the UPDRS III total score, the more severe the motor symptoms of PD patients.

Motor phenotypes, including tremor-dominant (TD), postural instability and gait difficulty (PIGD), and mixed types, were identified in PD patients according to Jankovic’s clinical phenotype classification [15]. Tremor was assessed by the item 16, 20, and 21, and PIGD by the item 13, 14, 15, 29, and 30 in UPDRS III. Motor phenotypes were determined by the ratio of the average tremor score to the average PIGD score, and the ratios of ≥ 1.5, ≤ 1.0, and 1.0-1.5 were identified as TD, PIGD, and mixed types, respectively.

#### Assessments of non-motor symptoms

A variety of non-motor symptoms were assessed by using the following rating scales during the “off” period to minimize the influence of medications on the assessments of non-motor symptoms [4], including Restless Legs Syndrome (RLS) Severity Rating Scale (RLSRS) for RLS, Rapid Eye Movement Sleep Behavior Disorder (RBD) Screening Questionnaire (RBDSQ) for RBD, Pittsburgh Sleep Quality Index (PSQI) for sleep quality, Scale for Outcomes in PD for Autonomic Symptoms (SCOPA-AUT) for autonomic dysfunction, 14-item Chalder Fatigue Scale (FS-14) for fatigue, and Mini-mental State Examination (MMSE) for cognitive impairment.

#### Assessments of activities of daily living (ADL)

ADL, including basic ADL (BADL) and instrumental ADL (IADL), were assessed by the 20 items of ADL scale [16].

### Measurements of free radicals, inflammatory factors, and pathological proteins in serum

#### Collection and treatment of serum samples

Under fasting condition, 2 ml of venous whole blood was collected from each patient in a polypropylene tube from 07:00 to 10:00. Then, serum sample was centrifuged immediately at 3000 rpm at 4 ℃. Finally, approximately 0.5 ml of serum supernatant was put into separate Nunc cryotubes (Beijing JingkeHongda Biotechnology Co., Ltd.) and frozen at -80 ℃. Freezing, thawing, and protein degradation were carefully avoided.

#### Measurements of free radicals in serum

The levels of free radicals, including H_2_O_2_, NO, and ·OH, in the serum from PD-A and PD-NA groups were measured by A018 kit, A064 kit, and A012 kit (Nanjing Jiancheng Biological Engineering Research Institute, Nanjing, China), respectively, by using chemical colorimetric method.

#### Measurements of inflammatory factors in serum

The levels of inflammatory factors, including TNF-α, IL-1β, IL-6, PGE_2_, and INF-γ, in the serum from PD-A and PD-NA groups were measured by using enzyme-linked immunosorbent assay (ELISA). 1R350 kit (Rapidbio Company, Shanghai, China), 1R040 kit (Beijing DOP Biotechnology Co., Ltd, Beijing, China), 1R140 kit (RB Company, Shanghai, China),, and CSB-E07965h kit (CUSABIO Company, Wuhan, China), and 1R330 kit (Rapidbio Company, Shanghai, China) were used for the measurements of TNF-α, IL-1β, IL-6, PGE_2_, and INF-γ, respectively.

#### Measurements of pathological proteins in serum

The levels of pathological proteins, including α-synuclein, Aβ_1−42_, P-tau (T181), P-tau (S199), P-tau (T231), P-tau (S396), and T-tau, in the serum from PD-A and PD-NA groups were measured by ELISA. CSB-E18033h kit, CSB-E10684h kit, and CSBE12011h kit (CUSABIO Company, Wuhan, China) were used for the measurements of α-synuclein, Aβ_1−42_,and T-tau, respectively. KHB7031 kit, KHO0631 kit, KHB7041 kit, and KhB8051 kit (Invitrogen Company, Carlsbad, America) were used for the measurements of P-tau (T181), P-tau (S199), P-tau (T231), and P-tau (S396), respectively.

### Statistical analysis

Statistical analyses were performed by SPSS Statistics 24.0 (IBM Corporation, New York, USA). The *P* value of less than an alpha level of 0.05 was defined as statistically significant.

The levels of free radicals, inflammatory factors, and pathological proteins in serum were compared between PD-A and PD-NA groups. Normally distributed measurement data were presented as mean ± standard deviation (SD), and non-normally distributed measurement data were presented as median (first quartile, third quartile). In the comparative analyses between PD-A and PD-NA groups, the data consistent with normal distribution and homogeneity of variance were analyzed by t test; while other data were analyzed by Mann-Whitney U test.

Binary logistic regression analyses were performed to identify the correlations among characteristics, motor phenotypes, and the levels of free radicals, inflammatory factors, and pathological proteins in the serum from PD patients. The dependent variables were anxiety and no anxiety rather than HAMA score, and the independent variables were free radicals, inflammatory factors, and pathological proteins in serum, age, gender, and UPDRSIII score. We used backward to enter independent variables, because we aimed to examine the relationship between all independent and dependent variables. In this model, omnibus test resulted in *p* = 0.006, and Hosmer-Lemeshow goodness-of-fit test resulted in *p* = 0.645.

Partial correlation analyses were performed to identify the correlation of HAMA-14 score with the levels of free radicals, inflammatory factors, and pathological proteins in the serum from patients with PD. A previous study showed that anxiety in PD patients could be affected by age, gender and disease severity. Thus, the results were adjusted for the above factors in order to reduce their influences [17].

## Results

### The frequency of PD-A

Among 121 patients with PD, 76 cases were diagnosed with anxiety, showing a frequency of PD-A up to 62.81%. This indicated that anxiety was a very common non-motor symptom of PD. Among those with anxiety, 45cases (37.19%) were mild or possible anxiety, 22 cases (18.18%) were moderate or positive anxiety, 6 cases (4.96%) were obvious anxiety, and 3 cases (2.48%) were severe anxiety.

### Demographic variables and clinical information of PD-A and PD-NA groups

The demographic variables, including gender, age, age of onset, disease duration, years of education, side of onset, and LEDD, of PD-A and PD-NA groups were compared. The results showed no significant differences in the above demographic variables between the two groups, indicating that these variables measured in this study were comparable (Table 1).

**Table 1:** Comparisons of demographic variables, clinical information, motor function, non-motor symptoms, and ADL between PD-A and PD-NA groups

	PD-NA group	PD-A group	*P*
	(45 cases)	(76 cases)	
**Demographic variables**			
Female (n,%)	18 (40.00%)	34 (44.74%)	0.612
Age (years, ® ± SD)	61.33 ± 9.29	58.68 ± 10.13	0.155
Age of onset (years, x̅± SD)	58.01 ± 9.64	54.62 ± 10.64	0.087
Disease duration [years, Median (Q1-Q3)]	3.00 (2.00–4.00)	2.50 (1.00–5.00)	0.999
Years of education (years, ® ± SD)	8.50 ± 4.49	9.34 ± 4.46	0.324
Side of onset (left) (n, %)	18 (40.00%)	34 (44.74%)	0.820
LEDD [mg, Median (Q1-Q3)]	0.00 (0-200.00)	0.00 (0.00-300.00)	0.231
**Motor function**			
**H-Y stage (n,%)**			0.414
Early stage (H-Y stage 1-2.5)	38 (84.44%)	61 (80.26%)	
Advanced stage (H-Y stage 3–5)	7 (15.56%)	15 (19.74%)	
**Total UPDRS III score**	15.00 (12.00-30.50)	24.50 (17.25–29.75)	0.009*
[points, Median(Q1-Q3)]			
Tremor	3.00 (2.00-6.50)	4.50 (2.25-7.00)	
Rigidity	2.00 (1.00-5.50)	5.00 (2.00–8.00)	
Bradykinesia	7.00 (4.00–13.00)	12.00 (7.00-16.75)	
Postural instability and gait difficulty	2.00 (1.00-4.50)	4.00 (2.00–5.00)	
**Motor phenotype (n,%)**			0.297
PIGD type	11 (24.44%)	22 (28.95%)	
TD type	6 (13.33%)	15.00 (19.74%)	
Mixed type	28 (62.22%)	39 (51.32%)	
**Non-motor symptoms**			
**Sleep**			
RLSRS	0.00 (0.00–0.00)	5.50 (0.00-18.25)	0.003**
[points, Median(Q1-Q3)]			
RBD (RBDSQ>5 points)	5 (11.11%)	21 (27.63%)	0.033*
(n, %)			
PSQI	4.00 (2.00–6.00)	8.00 (5.75-14.00)	<0.001**
[points, Median(Q1-Q3)]			
**Autonomic nervous system**			
SCOPA -AUT	32.00 (28.00-35.75)	35.00 (33.00–40.00)	<0.001**
[points, Median(Q1-Q3)]			
**Fatigue**			
FS-14	6.50 (3.00–10.00)	10.00 (7.50–12.00)	<0.001**
[points, Median(Q1-Q3)]			
Cognitive function			
MMSE	28.00 (24.00–30.00)	27.00 (24.00-29.50)	0.450
[points, Median(Q1-Q3)]			
**ADL**			
ADL	22.00 (20.00–26.00)	28.50 (22.00–41.00)	0.002**
[points, Median(Q1-Q3)]			

*P < 0.05, **P < 0.01

LEDD: levodopa equivalent daily dose; H-Y stage: Hoehn-Yahr stage; UPDRS III: Unified Parkinson’s Disease Rating Scale III; TD: tremor-dominant; PIGD: postural instability and gait difficulty; RLSRS: Restless Legs Syndrome (RLS) Severity Rating Scale; RBDSQ: Rapid Eye Movement Sleep Behavior Disorder (RBD) Screening Questionnaire; PSQI: Pittsburgh Sleep Quality Index; SCOPA-AUT: Scale for Outcomes in PD for Autonomic Symptoms for autonomic dysfunction; FS-14: 14-item Chalder Fatigue Scale; MMSE: Mini-Mental State Examination; ADL: the Activities of Daily Living

### Motor function, non-motor symptoms, and ADL of patients in PD-A and PD-NA groups

The scores of motor function, non-motor symptoms, and ADL were compared between PD-A and PD-NA groups. The results suggested that PD-A group had significantly higher scores of UPDRS III, RLSRS, PSQI, SCOPA-AUT, FS-14, and ADL scales than PD-NA group (*P* < 0.05) (Table 1). The number of patients with a RBDSQ score higher than 5 points (diagnosing with PRBD) in PD-A group was significantly higher than that in PD-NA group (*P* < 0.05)(Table 1).

### Comparisons of the levels of free radicals, inflammatory factors, and pathological proteins in serum between PD-A and PD-NA groups

The levels of free radicals, including ·OH, H_2_O_2_, and NO, in serum were measured and compared between PD-A and PD-NA groups. The results indicated that ·OH level in the serum from PD-A group was significantly elevated compared with PD-NA group (*P* < 0.05). NO level in the serum from PD-A group was significantly decreased compared with PD-NA group (*P* < 0.05).

The levels of inflammatory factors, including TNF-α, IL-1β, IL-6, PGE_2_, and INF-γ,in serum were measured and compared between PD-A and PD-NA groups. The results suggested that TNF-α level in the serum from PD-A group was significantly increased compared with PD-NA group (*P* < 0.05).

The levels of pathological proteins, including α-synuclein, Aβ_1−42_, P-tau (T181), P-tau (S199), P-tau (T231), P-tau (S396), and T-tau, in serum were also measured and compared between PD-A and PD-NA groups. The results revealed no significant difference in the levels of the above pathological proteins in serum between the two groups (*P* > 0.05) (Table 2).

**Table 2:** Comparisons of the levels of free radicals, inflammatory factors, and pathological proteins in serum between PD-A and PD-NA groups

	PD-NA group	PD-A group	*P*
	(45 cases)	(76 cases)	
**Free radicals**			
·OH (U/L, Median (Q1-Q3)]	434.41 (257.51–538.50)	611.40 (485.32-706.74)	<0.001**
H_2_O_2_ [mmol/L, Median (Q1-Q3)]	31.79 (23.07–45.35)	32.89 (25.93–44.19)	0.401
NO [mmol/L, Median (Q1-Q3)]	60.74 (44.73–80.26)	47.03 (34.99–64.30)	0.027*
**Inflammatory factors**			
TNF-α [pg/ml, Median (Q1-Q3)]	37.79 (22. 84-58.74)	63.06 (37.68–75.67)	0.001*
IL-1β [pg/ml, Median (Q1-Q3)]	15.73 (9.08–22.33)	14.24 (9.96–20.78)	0.910
IL-6 [pg/ml, Median (Q1-Q3)]	4.06 (1.76–8.37)	3.62 (2.00-6.19)	0.867
PGE_2_ [pg/ml, Median (Q1-Q3)]	7.57 (3.37–12.75)	8.70 (4.56–13.48)	0.247
INF-γ [pg/ml, Median (Q1-Q3)]	8.25 (4.10–11.00)	6.99 (3.57–9.97)	0.354
**Pathological proteins**			
α-synuclein [ng/ml, Median (Q1-Q3)]	54.63 (40.69–87.87)	58.79 (33.85–86.42)	0.849
Aβ_1−42_[ng/ml, Median (Q1-Q3)]	0.75 (0.36–1.23)	0.54 (0.30–1.11)	0.245
P-tau (T181) [ng/ml, Median (Q1-Q3)]	78.07 (48.63-105.33)	60.71 (41.34–98.62)	0.146
P-tau (S199) [ng/ml, Median (Q1-Q3)]	7.07 (4.80-10.77)	6.56 (5.35–8.52)	0.731
P-tau (T231) (ng/ml, x̅± SD)	157.34 ± 70.00	139.39 ± 61.74	0.190
P-tau (S396) [ng/ml, Median (Q1-Q3)]	61.91 (35.73–83.71)	59.52 (41.49–80.60)	0.731
T-tau (ng/ml, x̅ ± SD)	112.11 ± 57.55	95.83 ± 54.67	0.162

*P < 0.05, **P < 0.01

·OH: hydroxyl radical; H_2_O_2_: hydrogen peroxide; NO: nitric oxide; TNF-α: tumor necrosis factor-α; IL-1β: interleukin-1β; IL-6: interleukin-6; PGE_2_: prostaglandin E_2_; INF-γ: interferon-γ; Aβ_1−42_: β amyloid_1 − 42_; P-tau (T181): phosphorylated tau (T181); P-tau (S199): phosphorylated tau (S199); P-tau (T231): phosphorylated tau (T231); P-tau (S396): phosphorylated tau (S396); T-tau: total tau

### The association between PD characteristics, the levels of free radicals, inflammatory factors, and pathological proteins in serum, and anxiety in patients with PD

**Fig. 1:**
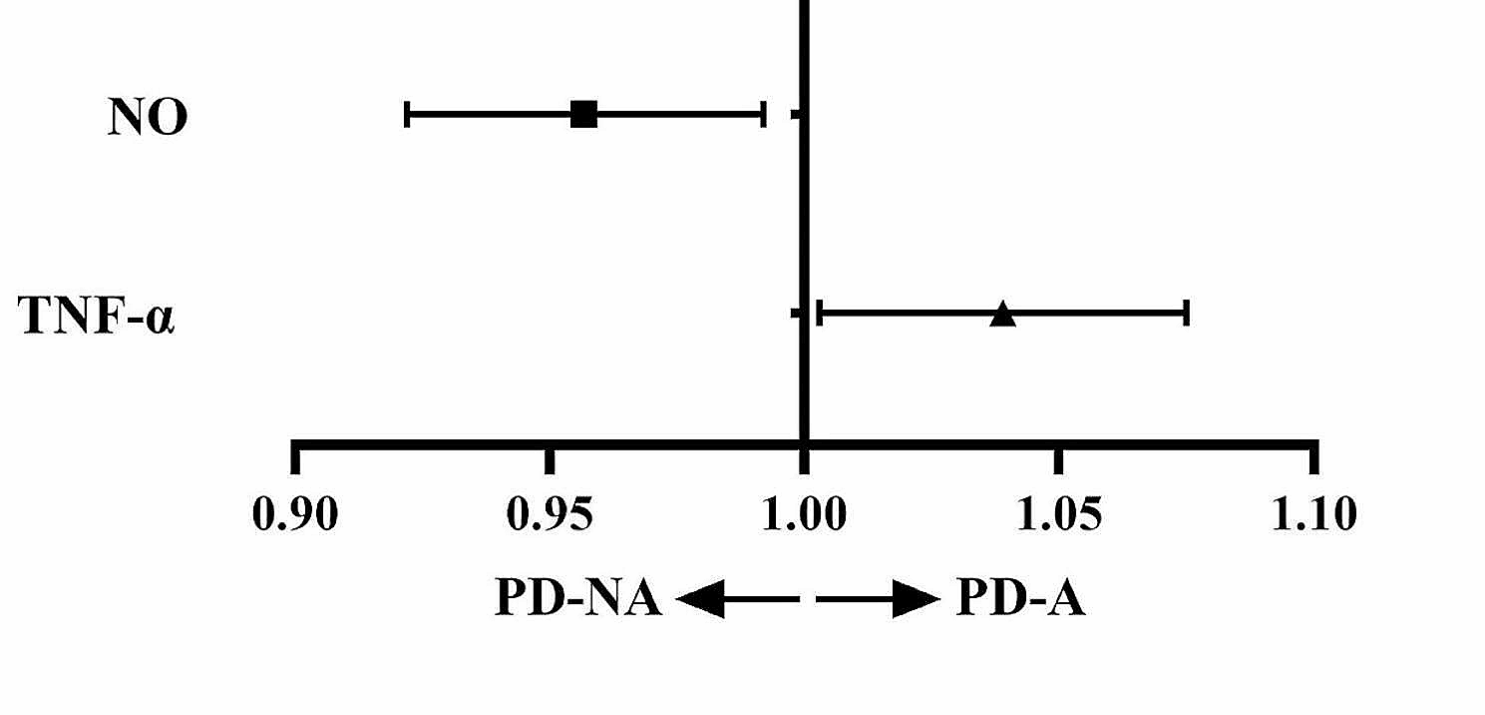
The levels of ·OH, NO, and TNF-α in serum and risk of developing anxiety in PD patients ·OH: hydroxyl radical; NO: nitric oxide; TNF-α: tumor necrosis factor-α

Binary logistic regression analyses showed that the levels of ·OH, NO, and TNF-α in serum were correlated with anxiety in patients with PD. There was no association of anxiety with UPDRS III score, motor phenotype, and the levels of other free radicals, inflammatory factors, and pathological proteins in the serum from patients with PD (Table 3; Fig. 1).

]

**Table 3:** Association between PD characteristics, the levels of free radicals, inflammatory factors, and pathological proteins in serum, and anxiety in PD patients

	OR	95%CI	*P*
** PD characteristics**			
Age	0.938	0.854–1.030	0.178
Sex	0.968	0.235–3.993	0.964
Total UPDRS III score	0.994	0.938–1.052	0.829
**Motor phenotype**			
TD type vs. Mixed type	1.300	0.239–7.077	0.761
PIGD type vs. Mixed type	4.169	0.516–33.656	0.180
**Serum free radicals**			
·OH	1.005	1.001–1.010	0.036*
H_2_O_2_	1.008	0.987–1.030	0.442
NO	0.956	0.922–0.992	0.017*
**Serum inflammatory factors**			
TNF-α	1.039	1.003–1.075	0.033*
IL-1β	0.977	0.912–1.047	0.511
IL-6	1.021	0.997–1.046	0.092
PGE_2_	1.144	0.975–1.344	0.100
INF-γ	0.999	0.902–1.108	0.990
**Serum pathological proteins**			
α-synulein	1.014	0.990–1.038	0.248
Aβ_1−42_	1.417	0.799–2.511	0.233
P-tau (T181)	0.982	0.960–1.005	0.119
P-tau (S199)	1.068	0.874–1.305	0.520
P-tau (T231)	0.994	0.984–1.004	0.228
*P*-tau (S396)	1.010	0.988–1.032	0.377
T-tau	0.995	0.983–1.007	0.429

*P < 0.05

UPDRS: Unified Parkinson’s Disease Rating Scale; ·OH: hydroxyl radical; H_2_O_2_: hydrogen peroxide; NO: nitric oxide; TNF-α: tumor necrosis factor-α; IL-1β: interleukin-1β; IL-6: interleukin-6; PGE_2_: prostaglandin E_2_; INF-γ: interferon-γ; Aβ_1−42_: β amyloid_1 − 42_; P-tau (T181): phosphorylated tau (T181); P-tau (S199): phosphorylated tau (S199); P-tau (T231): phosphorylated tau (T231); P-tau (S396): phosphorylated tau (S396); T-tau: total tau

### The correlation of HAMA-14 score with the levels of free radicals, inflammatory factors, and pathological proteins in the serum from PD patients

**Fig. 2:**
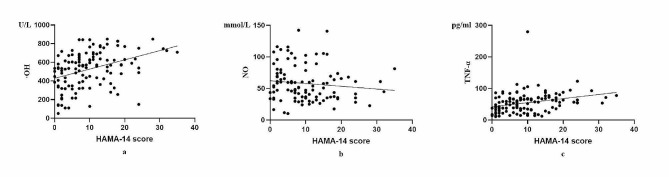
Correlations between the levels of ·OH, NO, and TNF-α in serum, and HAMA-14 score in patients with PD ·OH: hydroxyl radical; H_2_O_2_: hydrogen peroxide; NO: nitric oxide; TNF-α: tumor necrosis factor-α

The correlation of HAMA-14 score with the levels of ·OH, NO, and TNF-α in the serum from PD patients were analyzed. It was found that HAMA-14 score exhibited significantly positive correlation with the levels of·OH, and TNF-α in the serum from PD patinets (*P* < 0.01) (Table 4; Fig. 2).

**Table 4:** Correlation of HAMA-14 score with the levels of free radicals, inflammatory factors, and pathological proteins in the serum from PD patients

	R	*P*
**Free radicals**		
·OH [U/L, Median (Q1-Q3)]	0.358	<0.001**
H_2_O_2_ [mmol/L, Median (Q1-Q3)]	0.175	0.070
NO [mmol/L, Median (Q1-Q3)]	-0.143	0.153
**Inflammatory factors**		
TNF-α [pg/ml, Median (Q1-Q3)]	0.277	0.002**
IL-1β [pg/ml, Median (Q1-Q3)]	0.024	0.807
IL-6 [pg/ml, Median (Q1-Q3)]	0.067	0.549
PGE_2_ [pg/ml, Median (Q1-Q3)]	0.032	0.744
INF-γ [pg/ml, Median (Q1-Q3)]	-0.026	0.817
**Pathological proteins**		
α-synulein [ng/ml, Median (Q1-Q3)]	-0.049	0.613
Aβ_1−42_ [ng/ml, Median (Q1-Q3)]	-0.025	0.798
P-tau (T181) [ng/ml, Median (Q1-Q3)]	0.089	0.382
*P*-tau (S199) [ng/ml, Median (Q1-Q3)]	-0.097	0.339
P-tau (T231) (ng/ml, x̅ ± SD)	-0.004	0.972
P-tau (S396) [ng/ml, Median (Q1-Q3)]	-0.039	0.702
T-tau (ng/ml, x̅ ± SD)	-0.135	0.162

**P < 0.01, *P* values were adjusted for age, sex, and total UPDRS III score

·OH: hydroxyl radical; H_2_O_2_: hydrogen peroxide; NO: nitric oxide; TNF-α: tumor necrosis factor-α; IL-1β: interleukin-1β; IL-6: interleukin-6; PGE_2_: prostaglandin E_2_; INF-γ: interferon-γ; Aβ_1−42_:β amyloid_1 − 42_; P-tau (T181): phosphorylated tau (T181); P-tau (S199): phosphorylated tau (S199); P-tau (T231): phosphorylated tau (T231); P-tau (S396): phosphorylated tau (S396); T-tau: total tau

### The correlations between the levels of free radicals,inflammatory factors， and pathological proteins in the serum from PD-A and PD-NA groups

The correlations between the levels of free radicals andpathological proteins in the serum from PD-A and PD-NA groups was also analyzed.

**Fig. 3:**
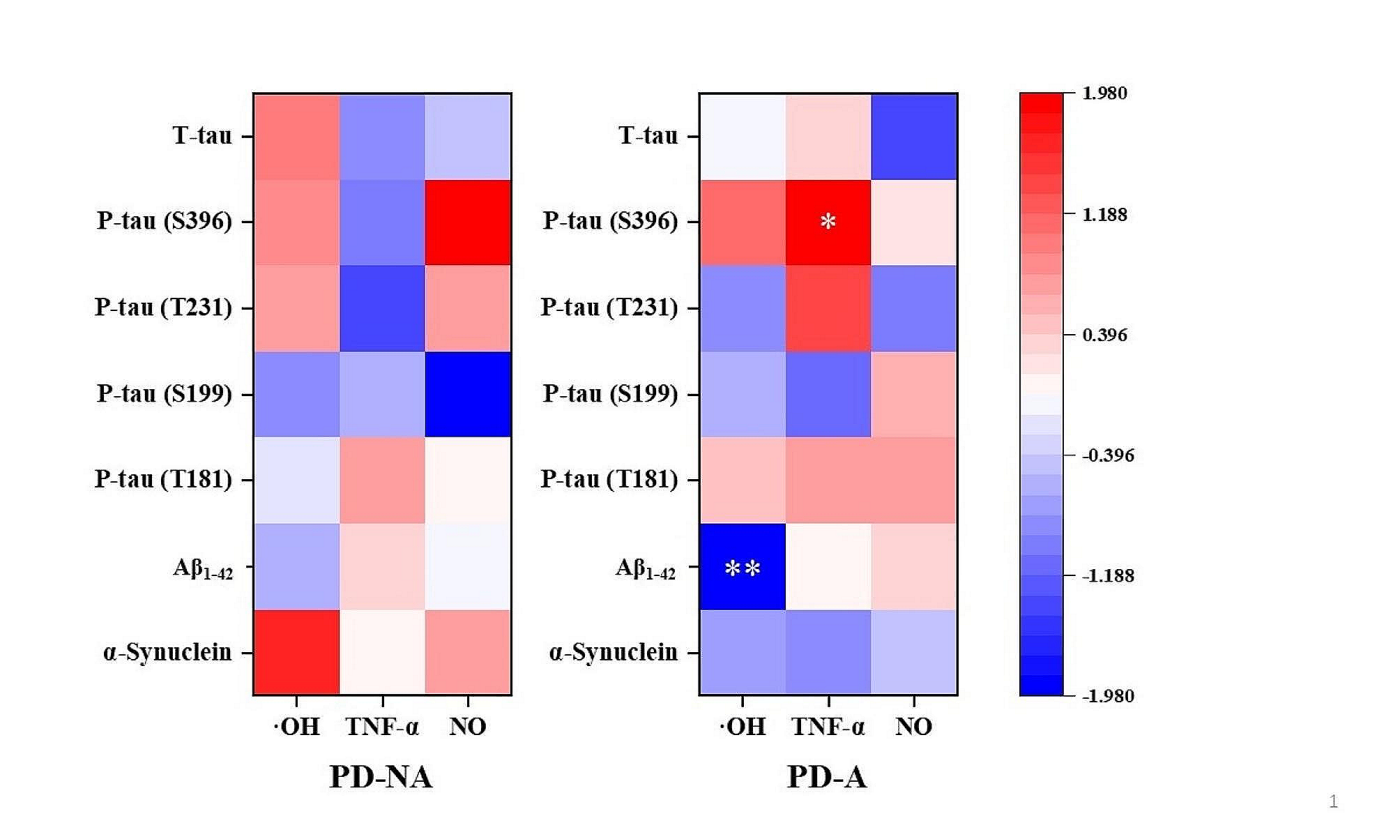
Correlations between the levels of free radicals, inflammatory factors, and pathological proteins in the serum from PD-A and PD-NA groups. *P < 0.05, **P < 0.01 ·OH:hydroxyl radical; TNF-α:tumor necrosis factor-α; NO: nitric oxide; Aβ_1−42_:β amyloid_1 − 42_; P-tau (T181): phosphorylated tau (T181); P-tau (S199): phosphorylated tau (S199); P-tau (T231): phosphorylated tau (T231); P-tau (S396): phosphorylated tau (S396); T-tau:total tau

In PD-A group, ·OH level exhibited a significantly negative correlation with Aβ_1-42_ level in serum (*P* < 0.01) (Fig. 3; Table 1). TNF-α level displayed a significantly positive correlation with P-tau (S396) level in serum (*P* < 0.05) (Fig. 3; Table 1). In PD-NA group, there was no significant correlation between the levels of free radicals, inflammatory factors, and pathological proteins in serum (Fig. 3; Table 2).

## Discussion

### Frequency of PD-A

Anxiety is one of the most common non-motor symptoms in PD patients. The incidence of PD-A ranged from 25% to 60% [1, 18–20]. Using a cut-off point of 13 for HAMA-14, the incidence of PD-A in China was 25.81% [20]. Based on the same cut-off value, the frequency of PD-A in this study was 26.4%. To identify patients with PD-A at the early stage of the disease, a more rigorous standard (a cut-off point of 7 for HAMA-14) was used, and the result was consistent with the previous finding.

### Motor function, non-motor symptoms, and ADL of PD-A and PD-NA groups

In this study, PD-A group manifested with severer motor symptoms (Table 1). As revealed in a previous study, PD patients complicated by severer motor symptoms, such as abnormal posture and gait, had a higher proportion of anxiety than those complicated by tremor, and the severity of dyskinesia was positively correlated with anxiety [21]. In this study, the patients in PD-A group also presented with more severe non-motor symptoms, such as sleep disturbances, autonomic dysfunction, and fatigue (Table 1).

Sleep disturbances may occur in patients with PD or anxiety. More complicated pathophysiological changes may appear under the existence of both conditions. Dopamine and 5-hydroxytryptamine (5-HT) could maintain wakefulness. With the progression of PD, the pronounced imbalance between dopamine and 5-HT caused severe sleep disturbances [22]. Most patients with RLS suffered from anxiety as well as depression [23]. The declined dopamine level was relevant to the occurrence and aggravation of RLS. In a previous study, it was found that PD patients with RLS had more severe motor symptoms and non-motor symptoms, such as anxiety, depression, fatigue, and apathy, than those without RLS. Dopaminergic disorders in central nervous system induced RLS, which aggravated anxiety in PD patients [24]. In previous studies, it was observed that PD patients with RBD experienced more severe anxiety than those without RBD. Interestingly, anxiety was related to the brain regions with impacts on RBD, such as locus coeruleus, and amygdala, which might explain the occurrence of more severe RBD in patients with PD-A. Locus coeruleus sends direct projections to autonomic nucleus and spinal cord, thereby controlling autonomic nerves and related functions. Furthermore, locus coeruleus was activated by anxiety, which led to sympathetic nerve excitement and parasympathetic nerve inhibition, causing a variety of autonomic symptoms [25]. It was revealed that 5-HT levels in both the serum and CSF from PD patients with fatigue were significantly decreased compared with those without fatigue [26]. Additionally, it was further revealed that the levels of iron and transferrin in the CSF from PD patients with fatigue were significantly increased [26]. Hence, abnormal iron metabolism might elicit oxidative stress and reduce 5-HT level, thus leading to the occurrence and progression of PD-A. Mild cognitive impairment (MCI) at the early stage and dementia at the late stageconstitute major cognitive impairments in PD patients. For PD patients, MCI remained stable for a long time [27], and it took about 10 years for these patients to develop dementia [28]. In this study, we did not find significant difference in the scores of MMSE and MoCA scales between PD-A and PD-NA groups,which might be because that the patients in the two groups suffered from PD at an early stage (H-Y stage 1-2.5, 84.44% VS. 80.26%), and there was no significant difference in the disease duration [3.00 (2.00–4.00) VS. 2.50 (1.00–5.00)].

In this study, PD-A group had significantly compromised ADL indicated by the increased ADL score compared with PD-NA group (Table 1). This might be attributed to the more severe motor and non-motor symptoms in PD-A group.

### Oxidative stress/inflammation and PD-A

Oxidative stress is one of the important pathogenesis of PD [29]. It was observed that the levels of free radicals were significantly increased in the serum and CSF from PD patients [30]. Dopaminergic neurons in substantia nigra are particularly vulnerable to the toxicity induced by free radicals produced during oxidative stress, and thus associated with the motor symptoms of PD. Although there are some studies on the relationship between oxidative stress and PD, it remains unclear whether oxidative stress is associated with PD-A. Inflammation in brain is another pivotal pathogenesis of PD, which is characterized by the robust productions of numerous inflammatory factors. It was observed that inflammatory factors were increased in the serum and CSF from PD patients [8, 31], which was related to the activated microglia in brain [32].

In this study, the levels of ·OH and TNF-α in the serum from PD-A group were significantly elevated compared with PD-NA group (Table 2). Furthermore, ·OH and TNF-α were the risk factors of anxiety for PD patients (Table 3; Fig. 1). Additionally, HAMA-14 score was significantly and positively correlated with ·OH and TNF-α levels in the serum from PD patients (Table 4; Fig. 2). These data demonstrated that PD patients with anxiety had intensified oxidative stress and inflammation. Furthermore, anxiety became increasingly prominent as the levels of·OH and TNF-α in serum were elevated.

As an important oxygen-consuming organ [33], brain contains a large number of oxygen molecules, which constitute the substrate for the formation of free radicals, including superoxide, H_2_O_2_, ·OH, and NO. It is recognized that ·OH has the strongest oxidative capacity among all free radicals, which induces severe damage to neurons from both functional and morphological levels [34]. TNF-α is an important inflammatory factor relevant to PD. It was reported that TNF-α levels in the serum and CSF from PD patients were drastically increased [35]. Microglial activation and TNF-α elevation were all observed in the substantia nigra of PD patients [36]. This implied that TNF-α produced by activated microglia might be involved in the occurrence of motor symptoms in PD patients. Moreover, the increased TNF-α level in serum was correlated with the non-motor symptoms of PD, including depression, cognitive impairment, and sleep disorders [37]. HAMA score was positively correlated with TNF-α level in the serum of PD patients [38]. In this study, the pivotal role of inflammation on PD-A was validated by the remarkbly elevated TNF-α level in serum.

In PD patients, NO was considered as one of reactive oxygen species. As revealed in a previous study, NO level in the serum from PD patients was increased compared with control group [39]. However, NO level in serum was decreased in patients with anxiety, and the elevated NO level alleviated anxiety symptoms in patients with anxiety disorders and other mood disorders [40]. However, no study elucidates the role of NO in patients with PD-A. In this study, the focus was placed on both PD and anxiety. It was found that PD-A group had a significantly lower NO level than PD-NA group (Table 2). Hence, NO was identified as not only an important molecule for oxidative stress/ inflammation but also a neurotransmitter that may regulate other neurotransmitters. In rat and mouse experiments, NO with different doses showed an anxiolytic effect when it was injected into body through intraperitoneal or intraventricular injection [41]. Therefore, a decrease of NO in serum may be an important mechanism for the occurrence and progression of anxiety in patients with PD-A. The results from this study were highly suggestive of the roles of ·OH and TNF-α on oxidative stress/inflammation in patients with PD-A. NO can be considered a factor of oxidative stress or neurotransmitter, ans its role as a neurotransmitter was more prominent in PD-A. It is necessary to conduct more animal experiments to verify its role on PD-A.

### Correlations among PD-A, oxidative stress/ inflammation, and pathological proteins in serum

Aβ_1−42_ and P-tau are the major components of neuritic plaques and neurofibrillary tangles, respectively. It was suggested from increasing evidence that Aβ_1−42_ was deposited in the brains of PD patients. Aβ_1−42_ and P-tau were found to be associated with cognitive impairment in PD patients [6, 42]. Besides, the low Aβ_1−42_ level in CSF might be of predictive significance for cognitive impairment in PD patients [43]. PET study revealed that Aβ_1−42_ deposition was significantly increased in PD patients with MCI and dementia [44].

In PD-A group of this study, ·OH level exhibited a significantly negative correlation with Aβ_1−42_ level in serum. In addition, TNF-α level was significantly and positively correlated with P-tau (S396) level in the serum from PD-A group compared with that from PD-NA group (Fig. 3; Table 1). However, in PD-NA group, there was no correlations between the levels of free radicals and inflammatory factors, and pathological proteins in serum (Fig. 3; Table 2). These data suggested that once patients with PD had anxiety, the intensified oxidative stress/inflammation would be highly associated with the increased deposition of pathological proteins related to cognitive impairment, and the significantly elevated depositions of pathological proteins might be detected earlier than the decline of cognitive function. As per previous studies, cognitive impairment was stable and even reversible if diagnosis and intervention were provided at the early stage of PD for patients. Hence, it was of great importance to identify those who were susceptible to cognitive decline in PD population. Furthermore, the results of this study suggested that it was necessary to periodically assess cognitive function and monitor the levels of Aβ_1−42_ and P-tau (S396) in the serum for patients with PD-A.

In this study, there was no significant difference in α-synuclein level in serum between PD-A and PD-NA groups, and α-synuclein level was not correlated with the levels of free radical/inflammatory factors in the serum from PD-A group. These results implied that α-synuclein might not be directly associated with anxiety of PD, hence, other mechanisms need to be investigated in the future.

## Conclusions

In summary, the incidence of PD-A is 62.81%. PD-A patients have more severe motor dysfunction, multiple non-motor symptoms, including sleep disorders, autonomic dysfunction, and fatigue, and impairedADL. The increase in ·OH and TNF-α and the decrease in NO are all associated with more severe anxiety in PD patients. The findings from this study may provide in-depth insights into the clinical characteristics of PD-A and theircorrelation with oxidative stress, inflammation, and pathological proteins. Drug development by targeting oxidative stress, inflammation and pathological proteins of AD may be promising for eradicating anxiety and related cognitive decline in PD patients.

### Limitations

There are limitations in this study. Firstly, there is a lack of serum samples from control subjects due to the difficulty in collecting serum samples from the elderly population without any other physical disorders. In addition, the levels of oxidative stress, inflammation, and pathological proteins in serum may not be completely consistent with their levels in CSF, and both serum and CSF samples will be collected for the comparisons, and cortical thickness and volume of the brain regions associated with anxiety will be evaluated in the future to further illustrate the conclusion from this study.Furthermore, the scales of motor, non-motor and ADL share similar items, thus, it is difficult to completely rule out the correlation and collinearity among these variables. In the further study, the samples should be inreased to perform multivariate correlation analysis to rule out the correlation and collinearity among multiple variables.Moreover, there are interactions among oxidative stress, inflammation, and pathological proteins, which may cause collinearity when comparing these variables.The interactions and cascades among these variables are very complex in the real world, therefore, more investigations on the sophisticated mechanisms are urgently needed.

## Data Availability

The datasets used and/or analysed during the current study are available from the corresponding author on reasonable request.
